# Global trends in the burden of non-Hodgkin Lymphoma: a comprehensive analysis from 1990 to 2045

**DOI:** 10.1007/s10552-026-02167-8

**Published:** 2026-04-22

**Authors:** Shuangling Wang, Jun Wang, Jianxiong Lin

**Affiliations:** 1 Department of Hematology and Oncology, The Second Affiliated Hospital of Shantou University Medical College, Shantou, 515041 Guangdong Province China; 2 Laboratory Department, The Second Affiliated Hospital of Shantou University Medical College, Shantou, China

**Keywords:** Non-Hodgkin lymphoma, Global burden, Age standardization, Sociodemographic index, Forecasting

## Abstract

**Purpose:**

To analyze global trends in the non-Hodgkin lymphoma (NHL) burden from 1990 to 2021 and forecast future trajectories from 2022 to 2045.

**Methods:**

Using data from the GBD 2021 Study, we extracted age-standardized incidence, mortality, and disability-adjusted life year (DALY) rates and estimated their average annual percent changes (AAPCs). Age-period-cohort models were constructed to assess temporal trends, and decomposition analysis identified drivers of burden changes. Future projections were generated using the Nordpred model.

**Results:**

From 1990 to 2021, the global incidence of NHL increased (AAPC = 0.53; 95% CI: 0.50–0.56), whereas mortality (AAPC = – 0.41) and DALY rates (AAPC = – -0.60) decreased. Aging accounted for 35% of incident cases, population growth accounted for 49%, and epidemiological changes accounted for – 14%. By 2045, projected cases, and deaths are expected to reach 985,820 and 437,933 respectively.

**Conclusion:**

The global burden of NHL continues to increase in terms of incident cases, whereas mortality and DALY rates show declining trends. Aging and population growth are the primary drivers. By 2045, NHL will still be a substantial public health challenge. High SDI regions should address workforce shortages and treatment costs, while low SDI regions need investments in early detection and infection control programs.

## Background

Non-Hodgkin lymphoma (NHL) constitutes approximately 90% of all lymphoid neoplasms and is characterized by significant geographic and socioeconomic variability in terms of its incidence, mortality, and associated disability-adjusted life years (DALYs) [1]. In recent decades, the absolute number of global NHL cases has risen, influenced by population growth, aging, improved diagnostic capabilities, and increased awareness. Mortality trends, however, present a divergent picture: regions with robust health care systems have witnessed declines attributable to advances in targeted therapies, such as Bruton's tyrosine kinase inhibitors and chimeric antigen receptor T-cell treatments [2]. In contrast, low- and middle-income countries continue to face elevated burdens, exacerbated by delayed diagnosis and limited access to novel therapies. This socioeconomic gradient is stark; regions with high sociodemographic index (SDI) values exhibit a high incidence alongside declining mortality, whereas regions with low and middle SDI values shoulder a disproportionate share of premature deaths, which are often linked to infectious etiologies and health care disparities [3–5]. In this study, the Global Burden of Disease Study 2021 (GBD 2021) dataset was used to analyze the temporal dynamics of the NHL burden across 204 countries from 1990 to 2021, and future trends were projected to 2045. Our aim was to elucidate the drivers of disease burden, identify high-risk populations, and inform equitable resource allocation for cancer control.

## Methods

### Data source and processing

Data on NHL incidence, mortality, and DALYs for 204 countries and territories (1990–2021) were extracted from the GBD 2021 Study [6]. The GBD estimates were derived using standardized modeling tools, including meta-regression of available data sources (e.g., vital registration, cancer registries), to generate internally consistent estimates for all locations. All rates were age-standardized using the GBD 2021 global reference population. NHL cases were defined by International Classification of Diseases, 10th Revision codes (ICD-10: C82-C86.6, C96-C96.9).

### Dynamic global burden of NHL from 1990 to 2021

The average annual percent change (AAPC) represents the average annual rate of change of a variable. It was calculated by fitting a regression line to the logarithm of the variable values over time and then exponentiating the slope of that line.

### Temporal trend analysis

The average annual percent change (AAPC) was calculated by fitting a joinpoint regression model to the log-transformed, age-standardized rates over time, summarizing the trend across the entire period.

To disentangle the effects of age, calendar period, and birth cohort, we employed age-period-cohort models. These models use intrinsic estimators or similar methods to address the nonidentifiability problem (cohort = period + age). Model fit was assessed using the Akaike information criterion (AIC), where a lower value indicates a better balance of model fit and parsimony [7]. Age-period-cohort analyses were conducted using the age-period-cohort package in R.

### Decomposition analysis

A discrete-time decomposition method was applied to quantify the contributions of population aging, demographic shifts, and epidemiologic transitions to the burden of NHL. This approach partitioned changes in incidence, mortality, and DALYs into components attributable to the following:

Aging: A proportional increase in disease risk due to population aging.

Population Growth: The expansion of susceptible populations.

Epidemiologic Changes: Shifts in incidence and mortality rates independent of age and demography.

### Future projections

Long-term disease trajectories from 2022 to 2045 were forecasted using the Nordpred Bayesian hierarchical model [8]. This method incorporates spatial autocorrelation and uncertainty propagation, generating predictions with 95% credible intervals through Monte Carlo simulations (10,000 iterations).

### Statistical analysis

All analyses were conducted in R Studio (version 4.4.2) using specialized packages: INLA (v24.05.011) for Bayesian modeling of age-period-cohort trends, forecast (v8.20) for time series projections, and ggplot2 (v3.4.2) for data visualization.

Statistical significance was defined as p < 0.05 (two-tailed). Sensitivity analyses were conducted to validate the robustness of the findings against variations in model assumptions.

## Results

### *Dynamic* global burden of NHL from 1990 to 2021

Between 1990 and 2021, the global age-standardized incidence rates of NHL increased (AAPC = 0.53%, 95% CI: 0.50–0.56). In contrast, age-standardized mortality (AAPC = − 0.41%) and DALY rates (AAPC = − 0.60%) significantly decreased (Table 1 and 2). This divergence underscores improvements in survival against a backdrop of rising case detection.

**Table 1:** Age-standardized incidence, mortality, and DALY rates of non-Hodgkin lymphoma by continent and subregion, 1990–2021

Continent	Subregion	Incidence Rate			Mortality Rate			DALY Rate		
		1990	2021	AAPC	1990	2021	AAPC	1990	2021	AAPC
Africa	Central Sub–Saharan Africa	3	3.2	0.23*	2.9	2.8	–0.02	89.6	87.3	–0.06*
	Eastern Sub–Saharan Africa	6.1	6.1	0.02*	5.9	5.5	–0.23*	192.4	171.6	–1.82*
	Southern Sub–Saharan Africa	3.8	6.2	1.52*	3.8	6.2	1.52*	107.9	154.8	1.11*
Americas	Western Sub–Saharan Africa	2.8	3.3	0.54*	2.8	2.8	0.54*	98.4	94.1	–0.17*
	Andean Latin America	7.5	20.2	3.29*	4.2	4.9	0.58*	159.1	144	–0.67*
	**Caribbean**	**7.3**	7.4	0.08*	4.5	3.7	–0.59*	84.4	128.1	–0.01
	Central Latin America	3.8	6.1	1.50*	2.7	3.1	0.44*	92.6	90.8	–0.98*
	Southern Latin America	5.9	6.8	0.51*	5.9	6.8	0.51*	135	101.8	–0.90*
	Tropical Latin America	4.2	5.2	0.62*	4.2	5.2	0.62*	96.3	83.1	–0.47*
	High–income North America	19.3	16	–0.63*	7.1	4.7	–1.34*	166.5	110.5	–1.54*
Asia	Central Asia	2.7	2.5	–0.16*	1.8	1.4	–0.82*	86.2	51.9	0.18*
	East Asia	3.3	5.5	1.67*	2.7	2.2	–0.66*	89.6	78.7	–0.40*
	High–income Asia Pacific	6.3	9.6	1.40*	3.5	3.4	–0.15*	135.3	80.3	0.24*
	North Africa and Middle East	3.7	5.4	1.22*	3.7	5.4	1.22*	98.5	79.1	–0.72*
	South Asia	2.8	3.4	0.68*	2.6	2.6	0.09*	85.8	79.8	–0.22*
	Southeast Asia	2.8	3.6	0.82*	2.6	2.7	0.08*	87.1	84.4	–0.11*
Europe	Central Europe	4.2	7.6	1.88*	2.6	3	0.42*	96.2	82.4	–0.23*
	Eastern Europe	4.5	7.9	1.93*	2	2.5	0.80*	100.7	82.4	–0.76*
	Western Europe	11.2	14.3	0.82*	5.1	4	–0.79*	127.7	94.5	–0.93*
Oceania	Australasia	13.9	16.6	0.55*	6.2	4.4	–1.19*	73.1	105	–1.14*
	Oceania	2.2	2.6	0.50*	1.7	1.8	0.18*	85.8	79.8	–0.22*
Global	–	6.1	7.1	0.53*	3.6	3.2	–0.41*	112	93.1	–0.60*

*p < *0.01; Rates are per 100,000 population; AAPC* = *average annual percent change*

**Table 2:** Age-standardized incidence, mortality, and DALY rates of non-Hodgkin lymphoma by SDI region, 1990–2021

Sub region		Incidence rRate			Morality rRate			Daly rRate	
	1990	2021	AAPC	1990	2021	AAPC	1990	2021	AAPc
High SDI	11.9	13.0	0.31*	5.1	4.0	–0.79*	140.0	97.7	–1.17*
High-middle SDI	5.2	7.4	1.13*	3.0	2.7	–0.32*	98.5	79.1	–0.72*
Middle SDI	3.3	5.1	1.42*	2.7	2.5	–0.13*	89.6	78.7	–0.40*
Low middle SDI	2.8	3.5	0.76*	2.5	2.7	0.21*	86.9	85.1	–0.06*
Low SDI	3.9	4.0	0.05*	3.8	3.5	–0.22*	134.4	113.9	–0.53*

*p < *0.01; Rates are per 100,000 population*

*AAPC* *average annual percent change*

#### Geographic Heterogeneity in 2021

**Fig. 1:**
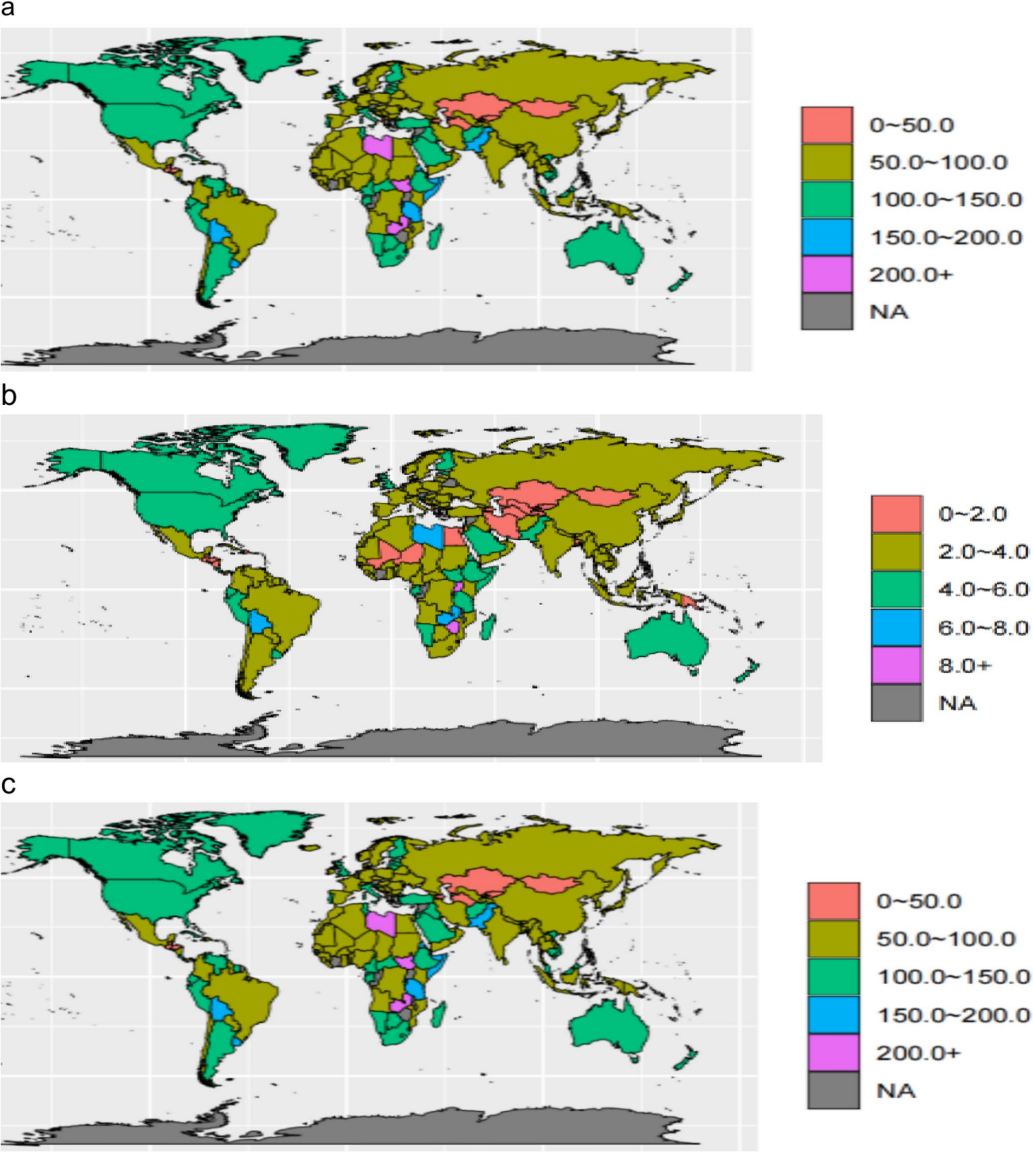
Geographic distribution of the NHL burden in 2021. **a** Age-standardized incidence rate. **b** Age-standardized mortality rate. **c** Age-standardized DALY rate

Considerable geographic disparity existed in 2021 (Fig. 1a–c). High SDI regions, such as Peru (24.0 per 100,000) and Slovenia (23.8 per 100,000), reported the highest age-standardized incidence rates. The lowest rates were observed in low SDI regions such as Kiribati (0.8 per 100,000). The mortality patterns revealed an inverse relationship; the highest age-standardized mortality rates were reported in low SDI countries such as Malawi (13.0 per 100,000) and Zimbabwe (9.5 per 100,000). The burden of disease, measured by DALY rates, was also highest in Malawi (426.1 per 100,000).

### Age-period-cohort analysis

**Fig. 2:**
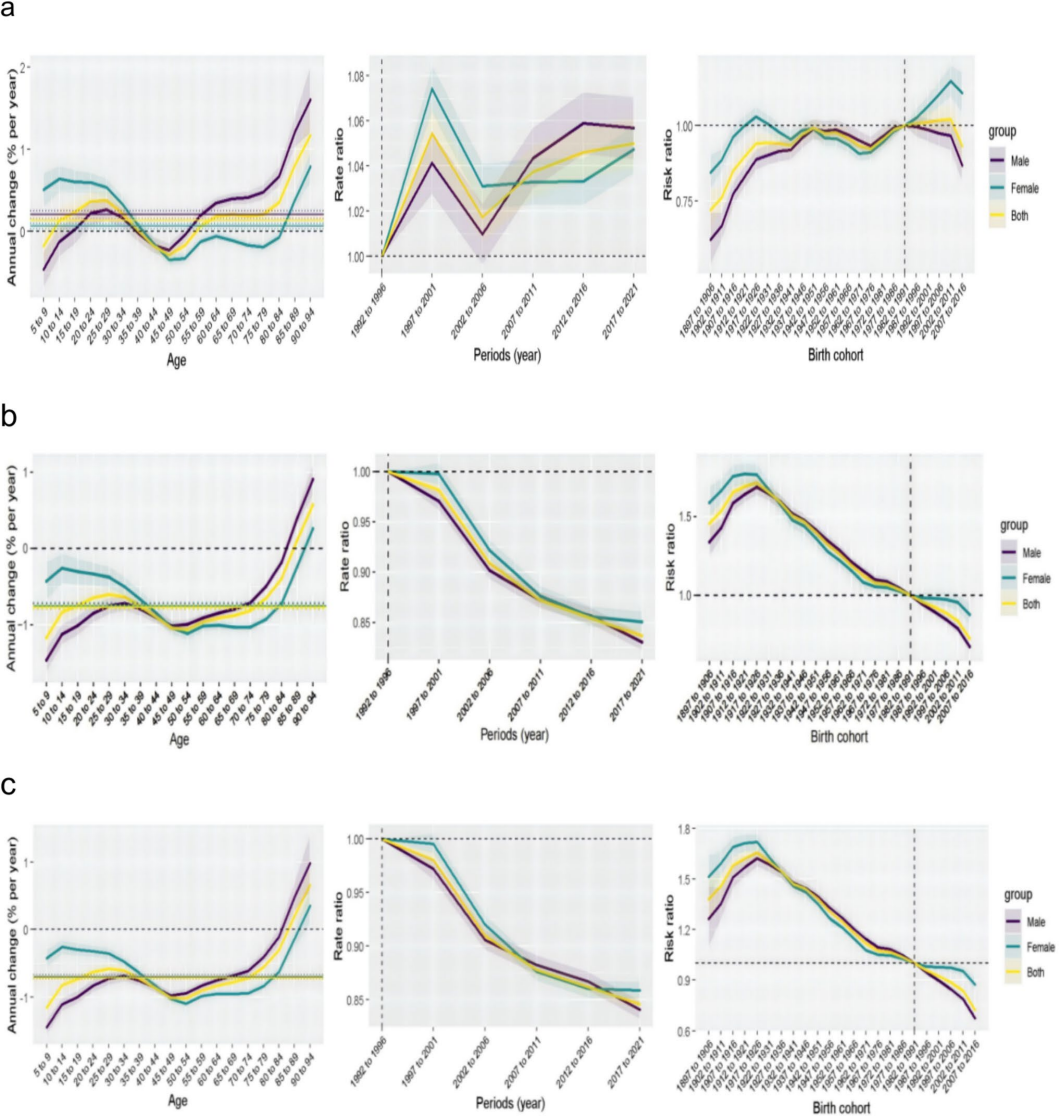
Age-period-cohort analysis of NHL burden, 1990–2021. Net effects for **a** incidence, **b** mortality, and **c** DALYs. Left panels: Age effects. Middle panels: period risk ratios (RR). Right panels: cohort RR. The reference lines are set at 1.0

Age-period-cohort analysis modeling revealed distinct underlying dynamics (Fig. 2a–c). The age-specific incidence showed a biphasic pattern, with rates rising steeply after middle age. Period effects indicated a steady increase in incidence risk ratios from the 1990s onward, whereas mortality and DALY risk ratios declined consistently over the same period. Cohort effects suggested that birth cohorts after the 1980s experienced higher incidence risk ratios, particularly among women, whereas mortality risk ratios declined monotonically across successive birth cohorts.

### Drivers of changes in absolute burden (1990–2021)

**Fig. 3:**
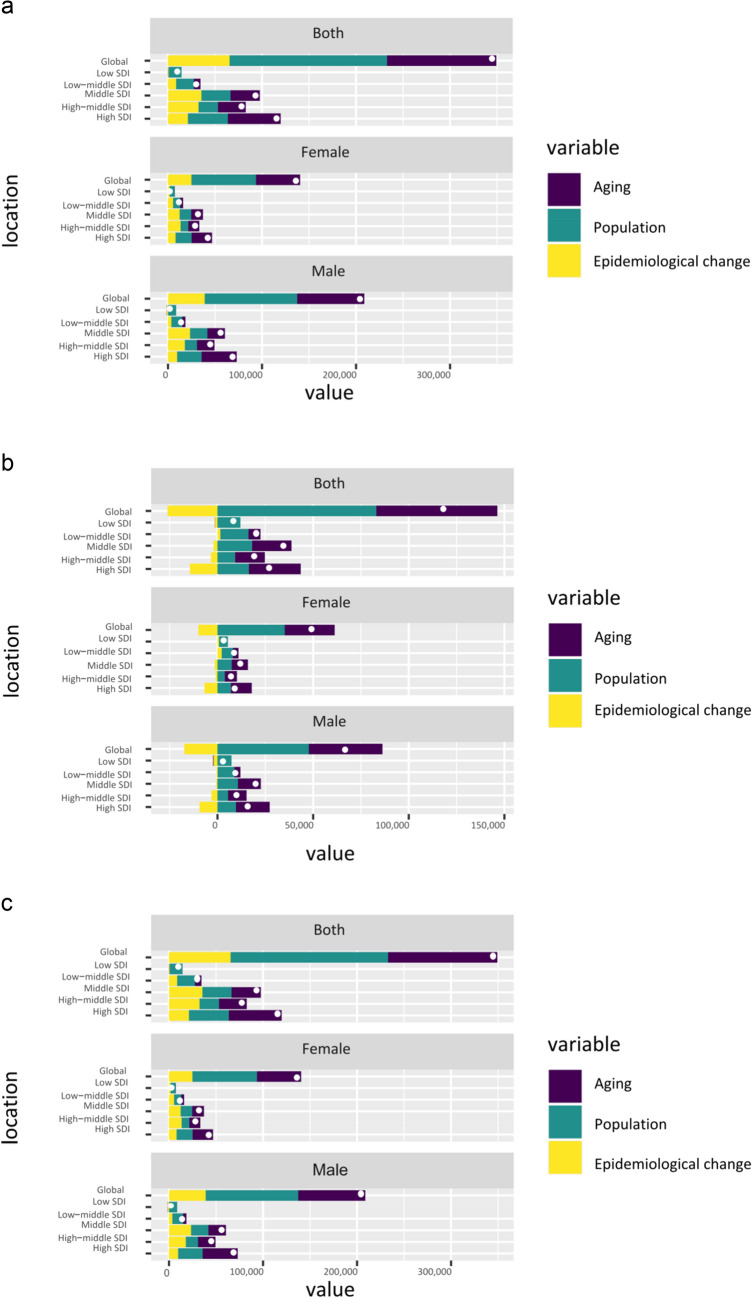
Decomposition of changes in the absolute burden of NHL, 1990–2021. Contributions of population growth, aging, and epidemiologic change to changes in **a** incident cases, **b** deaths, and **c** DALYs. Note: y-axes display absolute numbers.the dot within each bar indicates the net increase in the absolute NHL burden after accounting for the decrease attributable to positive epidemiological changes

Decomposition analysis quantified the drivers of change in absolute case numbers (Fig. 3a–c). Between 1990 and 2021, the increase in global incident cases was driven primarily by population growth (contributing 49%) and population aging (contributing 35%). Changes in epidemiology (e.g., risk factor dynamics, improved diagnostics) attenuated this increase by 14%. For mortality and DALYs, the favorable epidemiologic changes were almost entirely offset by demographic forces, resulting in stable or slightly increasing absolute numbers despite decreasing rates.

### Projections to 2045

**Fig. 4:**
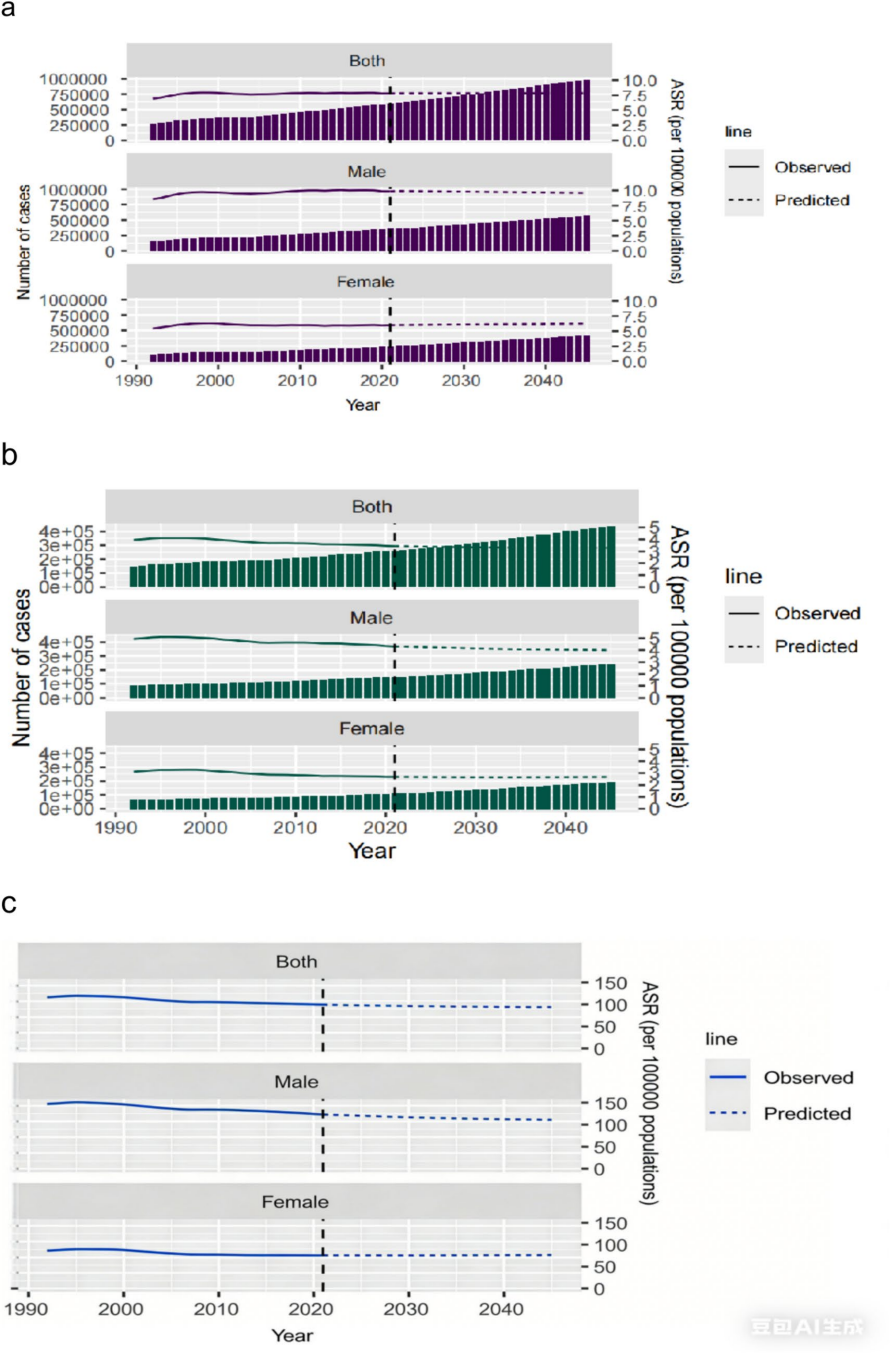
Observed (1990–2021) and projected (2022–2045) global burdens of NHL. **a** Incident cases. **b** Deaths. **c** DALYs. Shaded areas represent 95% credible intervals for projections

Projections indicate a continued increase in the absolute burden of NHL (Fig. 4a–c). By 2045, the annual number of new cases is projected to reach approximately 985,820, nearly double the 2021 estimate. The annual number of deaths is also projected to remain high, at approximately 437,933. ASR of DALYs is projected to decrease by 5.1 from 2021 levels.

## Discussion

This comprehensive analysis reveals the dual narrative of the global NHL burden: successful mitigation of mortality rates in many regions, juxtaposed with a relentless rise in absolute cases and persistent, severe inequities. While innovations such as CAR T-cell therapy and BTK inhibitors have transformed outcomes in high-resource settings, their global impact on mortality and disability remains attenuated by demographic momentum and disparities in access.

The observed increase in incidence is multifactorial. Beyond demographic shifts, enhanced detection through advanced imaging (e.g., PET-CT) contributes, although this may also introduce diagnostic inflation in trend interpretation [9]. The declining mortality rates in high SDI regions are a testament to therapeutic progress, yet the stasis or increase in mortality rates in low SDI regions underscores a systemic failure in equity.

The stark geographic disparities highlighted, such as the 18-fold difference in mortality between Peru and Malawi, cannot be explained by biology alone. They reflect profound inequities in health care infrastructure, diagnostic delays, and access to effective therapy [10–14]. Our decomposition analysis quantitatively underscores that without accelerated epidemiologic improvement (through prevention, early diagnosis, and equitable treatment), demographic forces will continue to drive the absolute burden upward, as confirmed by our projections to 2045.

## Limitations

Our findings must be interpreted within the constraints of the GBD modeling framework. While the GBD provides unparalleled comparability across time and location, its estimates are model-based reconstructions, particularly for incidence in regions with sparse cancer registry data [6]. Estimates for cancers with improved survival, such as some NHL subtypes, may have greater uncertainty. Furthermore, the projection model assumes that recent trends will continue, which may not account for future breakthroughs or systemic shocks. Only viral agents are mentioned among the NHL risk factors, whose control contributes to reducing the increasing number of cases (i.e., the burden) due to population aging. Although to a much lesser extent, the regulation of obsolete chemicals (pesticides, solvents, sterilizing agents) also contributes to reducing the number of cases. These limitations necessitate cautious interpretation of local policy but do not undermine the central findings regarding global trends and inequities.Future studies could further validate our findings by comparing GBD estimates with data from the IARC Globalcan database, particularly for regions with high burden disparities (e.g., Sub-Saharan Africa vs. high-income North America). This cross-validation would strengthen the robustness of causal factor identification (e.g., viral vs. chemical drivers) and inform more precise control resource allocation. Caution is warranted when using model-derived estimates to design local control programs, as unmeasured regional factors (e.g., unreported chemical exposures) may influence burden trends.

## Conclusions

From 1990 to 2021, the global NHL landscape shifted, with incidence rates increasing and mortality rates decreasing; however, the absolute burden increased because of population aging and growth. Projections to 2045 forewarn of a substantial and increasing public health challenge. Addressing this will require dual-pathway strategies: high SDI regions must prepare health care systems by increasing capacity and relieving financial pressures, while the international community must prioritize closing the equity gap in low SDI regions through investments in early detection, control of associated infections (e.g., EBV and HCV) [11, 12], and, crucially, sustainable access to effective therapies.

## Data Availability

The datasets generated and/or analyzed during the current study are available from the Global Health Data Exchange query tool (http://ghdx.healthdata.org/gbd-results-tool).
